# Spatio-Temporal Differences in Economic Security of the Prefecture-Level Cities in Qinghai–Tibet Plateau Region of China: Based on a Triple-Dimension Analytical Framework of Economic Geography

**DOI:** 10.3390/ijerph191710605

**Published:** 2022-08-25

**Authors:** Huasheng Zhu, Duer Su, Fei Yao

**Affiliations:** 1 Beijing Key Laboratory of Environmental Remote Sensing and Digital Cities, Beijing Normal University, Beijing 100785, China; 2 Faculty of Geographical Science, Beijing Normal University, Beijing 100785, China; suduer@mail.bnu.edu.cn (D.S.); feiyao1991@mail.bnu.edu.cn (F.Y.)

**Keywords:** regional economic security, triple-dimension analysis, economic geography, Qinghai–Tibet Plateau region

## Abstract

The assessment of regional economic security (RES) is mainly based on the theoretical ideas of political economy and marginalism, and the research areas are mainly concentrated in European and American countries/regions, especially Eastern Europe. Taking the Qinghai–Tibet Plateau in China as an example, this paper constructs a triple-dimensional analytical framework, resources, and environmental–economic foundation-driving forces, based on the institutional approach of economic geography, with the purpose of making up for the deficiency of the extant literature, which pays little attention to regional characteristics and the dynamic mechanism concerning RES, and to provide a tool to identify key factors affecting RES. This paper obtained the main conclusions as follows. (1) The index of the economic security in the Qinghai–Tibet Plateau is on the rise, and the difference at the level of RES among cities is significant but tends to decrease. (2) There is a significant spatial autocorrelation among cities in the Qinghai–Tibet Plateau in terms of RES. The high-value areas are concentrated along the southeast edge, and the low-value areas are concentrated in the central areas of the west. (3) Despite lower weight values, the weakness of the economic foundation and the fragility of the ecological environment has increasingly hampered the improvement of the economic security in the Qinghai–Tibet Plateau. In terms of driving forces, it is the support of the central government and aid programs of other provinces that contributes to its economic development.

## 1. Introduction

The term ‘economic security’ is used to describe a state in which a national economy is allowed to maintain resistance to both internal and external threats [1]. It also refers to the ability of a country to resist destructive actions and to maintain the sustainable development and competitiveness of the country in the global economic system [2]. As an integral part of national security, economic security has always been of concern to sovereign countries, and its importance has become increasingly prominent in the context of economic globalization. The 2008 financial crisis hit markets, finance, industry, and employment in countries around the world, and the economic security of countries was seriously threatened [3,4]. The outbreak of COVID-19 has led to increased risks in the global business environment [5,6], and countries have taken strategic preventive measures to enhance their economic security.

Typically, economic security has been discussed in the context of national and transnational regions; however, regional economic security (RES) within a country has become a heated topic recently. On the one hand, sub-national regions have been increasingly integrated into the global economic system and have been directly exposed to many uncertainties from outside, which interact with their internal factors and, jointly, have an impact on regional economic development. On the other hand, as an indispensable part of national economic security [7], RES may have a profound impact on the whole country, and the risks posed to a region by the cross-border network of non-state entities and the global environment may spill over into national economic security [4]. Inappropriate social-economic growth of regions may cause and further strengthen negative processes in the economic complex, which affects the stability of the economy and the sovereignty of a nation [8]. Under particular circumstances, the economic security of a specific region is of special significance to national security. For example, as the three most developed urban agglomerations in coastal areas of China, the Beijing–Tianjin–Hebei, Yangtze River Delta, and Pearl River Delta regions occupy a high position in the national economic security. The internal and external threats to the RESs of these three regions are especially important to the whole country [9]. Moreover, there is a large gap between the underdeveloped and developed areas in China. Provincial economic disparity was proven to have a negative effect on economic growth in China, which could limit the healthy development of the national economy or even increase social instability [10]. This is one of the main reasons why China’s central government has implemented a balanced regional development policy since the late 1990s. Particularly, border areas are sensitive areas for geopolitical, international trade, and economic and social issues and deserve much attention, especially for the border areas of multi-ethnic countries, whose socio-economic system and national security have a more complex interaction and function [11,12]. Extant literature mainly follows the theoretical ideas of political economy and marginalization [13] and focuses on the assessment of RES, while it ignores the characteristics of specific regions [7]. Furthermore, the research is mainly concentrated on a couple of countries, such as Russia and Ukraine, except for a few studies concerning the United States, Canada, the European Union, and Southeast Asia. There is a need for diverse cases, especially from countries such as China, where scholars have discussed RER topics domestically [14].

Therefore, the main purpose of this paper is to establish an assessment framework of RES by using the idea of interaction between humans and land in geography, paying attention to regional characteristics and differences, and using the institutional approach of economic geography, which is helpful for identifying and evaluating the internal and external threats that affect RES.

**Figure 1:**
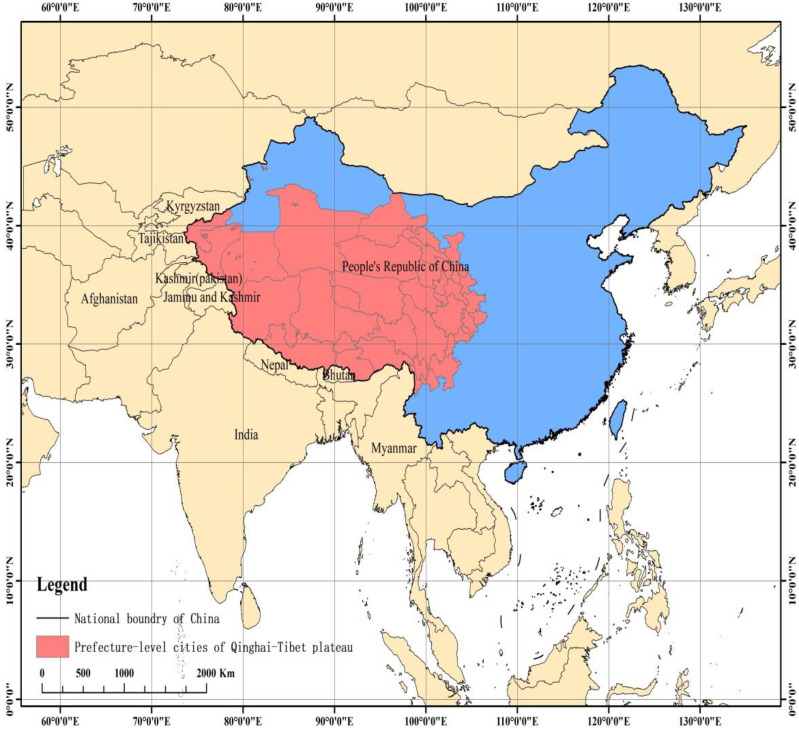
Sketch map of the study area.

**Figure 2:**
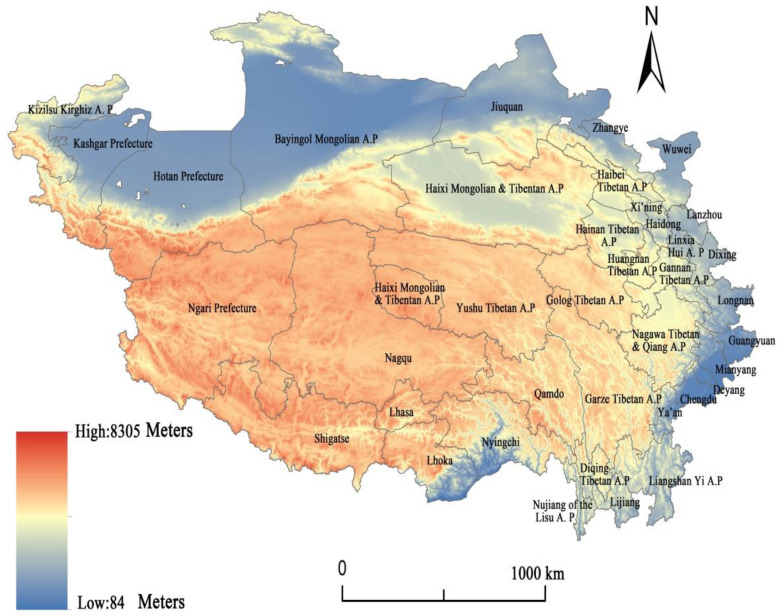
Elevation map of the study area. Note: The white spots on the map represent administrative units in the prefecture-level cities that do not belong to the Qinghai–Tibet Plateau region. The maps below are similar.

This paper takes China’s Qinghai–Tibet Plateau as the research area and provides means and tools for identifying key areas and important factors in regional security governance and facilitating public decision-making processes regarding appropriate investment, economic development, and ecological protection. The Qinghai–Tibet Plateau is a typical ethnic area of western China with an important geostrategic position, bordering Nepal, India, Bhutan, Pakistan, Afghanistan, Tajikistan, Kyrgyzstan, and other countries in southern and western Asia (Figure 1). It also has the following features. First, its ecosystem is unique and fragile. As the ‘Roof of the World’, the ‘Water Tower of Asia’, and an important ecological shelter zone in China and Asia, the Qinghai–Tibet Plateau is characterized by high altitude, thin air, strong solar radiation, complex topography (Figure 2), and complicated climatic conditions [15,16]. It is one of the regions greatly affected by global climate change, and the consequent reduction of glaciers and deterioration of the ecological environment (https://www.mfa.gov.cn/ce/cgcc/chn/kj/t576794/ accessed on 25 August 2022) will ultimately affect the regional and national security of China, and cross-border security in South Asia, Southeast Asia, and East Asia.

Second, due to the fragility of the ecological environment, the intensity of economic activities in the Qinghai–Tibet Plateau is less than in coastal and central areas of China; however, increasing development activities have a strong impact on the fragility of the ecological environment. Specifically, in addition to the pillar industries of traditional agriculture and animal husbandry oriented to local markets and its neighboring areas, tourism is a burgeoning pilot industry, with an increasing number of domestic and foreign tourists. Energy industries (such as wind power, solar power, and hydropower construction), non-ferrous metal mining and smelting, and chemical industry (such as oil and gas, chemical industry, crude salt, potassium fertilizer, and other salt chemicals production) have become the dominant industries in the domestic market and even satisfy global demands [17]. Some evidence shows that intensive development increases the tension between people and land and intensifies the conflict between man and beast [18,19]. Surely, different markets have different effects on different economic activities and ecological vulnerability, and consequently, RES. The interconnections between these metrics and the effect of changes in market composition over time deserve exploration.

Third, due to the harsh living environment and weak economic foundation, the Qinghai–Tibet Plateau was once a region with a relatively high incidence of poverty in China [20]. Such adverse economic conditions carry the risks of social unrest, which in turn worsens RES or regional security. Research shows that the riots and separatist activities in Tibet from 1987 to 1989 led to a significant decline in GDP growth potential [21]. As a developing ethnic area, its economic stability is crucial to the stability of social development and, consequently, regional security.

## 2. Literature Review and Analytical Framework of RES

### 2.1. Literature Review

As mentioned above, the research on economic security mainly focuses on nations and supranational regions, identifying and evaluating key internal and external risk factors and facilitators [22,23,24], with a theoretical basis in political economy and the school of marginalism [25]. Recently, academic interest has been extended to the issue of RES within a country, especially in Russia and Ukraine. This is because the drastic changes in the political and economic structure in Eastern Europe (including the disintegration of the Soviet Union) have increased the concerns about RES on the scale of subnational regions in the two countries [26]. Attention has been paid to the assessment of the status of RES and the identification of threats or crises. Table 1 lists some typical literature from recent years according to its research countries/regions, purposes/contributions, main ideas, assessment indicators, and methods, from which we can see the following characteristics.

First, the factors concerning economic security are diverse and not unique and are not confined to the economic dimension. They involve not only economic growth capacity but also regional political and socio-economic contexts [23,29], innovative development capacity [28], natural and resource potential [30], ecological environment quality, population and social development capacity [2], and other aspects. There are also significant differences in the indicator selection and the composition of the RES index due to different assessment objectives. For example, Gryshov et al. selected indicators that accord with the connotations of economic security from internationally renowned indexes such as the global competitiveness index, globalization index, fragile states index, Legatum prosperity index, human development index, and environmental performance index, and calculated the comprehensive economic security index by using geometric mean [2]. Kravchenko et al. emphasized bid-related indicators as the most important factor affecting the economic security of different regions, with the purpose of providing suggestions for promoting public services [22]. However, Lee is more concerned with the flexibility and depth of trade agreements between the Association of Southeast Asian Nations (ASEAN) and ASEAN countries, which is considered important to evaluate the economic security of Southeast Asia [24]. Osberg et al. mainly pay attention to citizens’ anxiety about economic crisis and welfare, adopts four key economic risks named in Section 25 of the Universal Declaration of Human Rights, and construct the RES index based on citizens’ human rights, integrating IEWB (Index of Economic Well-Being) security index, unemployment, illness, single-parent families, aging, etc. [31].

Secondly, the factors that affect economic security come from both inside and outside a region. The internal factors are represented by indicators including GDP growth rate [23,29], inflation rate [29,30], family saving and asset accumulation [32], employment or unemployment rate [2,30,31], industrial structure [29,33], government procurement of public services [22], investment in fixed assets [23], residents’ income [2,30], etc. The external factors mainly reflect the dependence of a country/region on the global economy or its competitiveness in the global economic system, which involves foreign investment and import and export trade, and some specific indicators, including the proportion of FDI (Foreign Direct Investment) in GDP [31,33], import index [23,31], export index [29,33], cross-border investment [24], foreign trade [30], foreign debts and liabilities [23], as well as comprehensive indicators reflecting the level of globalization, such as globalization index, global competitiveness (e.g., the effective integration of countries in the global value chain and their competitive advantages in key economic fields [2]), and global cooperation (e.g., the framework of regional economic cooperation and integration dealing with traditional and non-traditional issues [24]).

Thirdly, a variety of methods have been used to calculate the economic security index. Some research focuses on the quantitative level and dynamic changes of the indicators of economic security and the relationship between these indicators. For example, in Russia, Kravchenko et al. used least square regression to examine the relationship between different sub-components [22], and Karanina and Kartavyh analyzed the strengths and weaknesses of the economic security sub-system with SWOT analysis [29]. The IEWB index was used in research in North America [31] and provided a tool for identifying specified regional characteristics. Arithmetic progression was used in research in the European Union to study the difference in each index between different years and analyze their dynamic changes [23]. Other researchers have focused on the calculation of the index of economic security, which is conducive to judging the trend of economic security and identifying unsafe areas and is also convenient for evaluating the influence of indicators on economic security. The typical methods include the integral and weighted sum method [2,30] and fuzzy modeling in research in Ukraine [13], and the weighted method in research in China [14]. Additionally, the weighted sum method [27,30], cluster analysis [2,13], regression analysis [2,22], and integral analysis [9,30] are also used to examine the causal relations.

Generally speaking, despite the great differences in RES assessment systems, the majority of discussions or assessments still used the analytical framework of economic security on the scale of nations [13], namely, with the perspective of political economy and the school of marginalism [25], and did not develop a relatively unified system of RES indicators [34]. Indicator selection was mainly based on the macroeconomic level as well, covering many different metrics [13], while the characteristics and key factors of specific regions were ignored in most cases [34] except for a few studies [31]. Furthermore, as Chistnikova et al. (2017) advocated, in the analysis of RES, much attention should be paid to the basic conditions of regional economic development at present and the dynamic mechanism of its sustainable development in the future.

### 2.2. Analytical Framework of RES Indicator System

**Figure 3:**
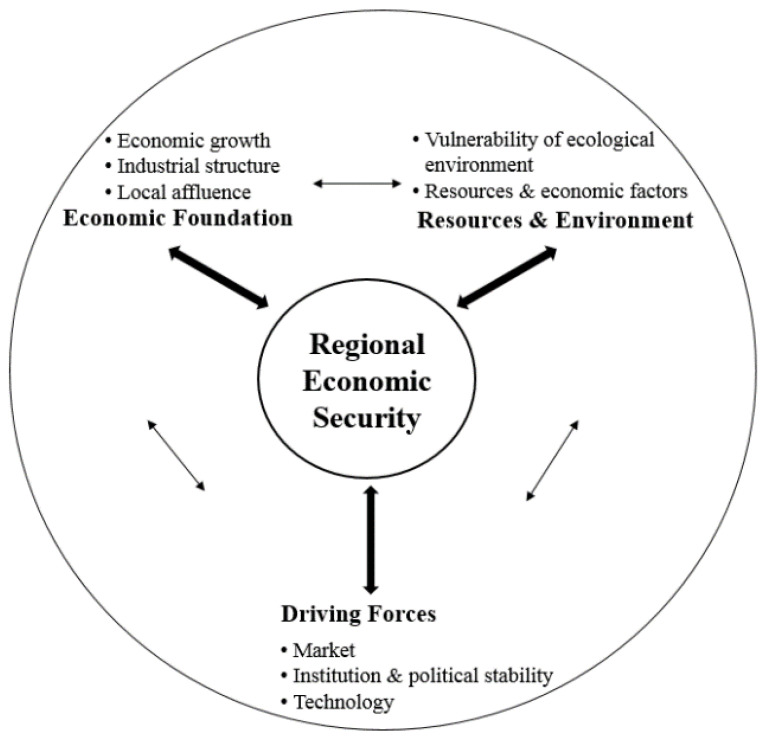
Analytical framework of RES assessment.

From the perspective of economic geography, this paper constructs an assessment indicator system of RES from the three dimensions of a region, namely, resources and environment (RE), economic foundation (EF), and driving forces (DF), aiming to enrich the theoretical basis of RES assessment. The analytical framework is shown in Figure 3.

First of all, RE constitutes the regional context of economic development and economic security. Ecology is a key component of national and regional economic security [2,30]. A few researchers have noticed the influence of RE on national economic security and have classified it as an ecological or environmental development factor [2]. According to the theory of the man–land relationship in geography, the natural environment is the material source of human survival and development, and it affects the development of social and economic activities through the social production mode [35]. The fragility of the environment limits the development intensity of regional resources and severely restricts the development of agricultural production activities, such as grain production [36], extractive industries, and other production activities, and consequently, economic growth [37]. In the face of major natural disasters and external shocks, economic development tends to stagnate and decline. The regional basic factor resources (such as labor resources, food resources, etc.) are not only the basic conditions for regional economic development but also affect the foreign trade dependence and the sustainability and stability of international cooperation of a country [38,39]. The relationship between food security and political risk [40] is an example.

Secondly, the foundation of economic development, such as regional affluence, economic structure, and growth rate, are necessary and essential for a region to resist internal and external risks and threats [27]. Regional affluence reflects residents’ income level, capital accumulation, and potential spending power, which can provide local spending power in the face of an external market downturn in the future. As part of human capital, health level affects an individual’s unemployment risk [41,42], the level and stability of his/her household income [43], expenditure on human capital, and other aspects of human well-being [44], which consequently becomes a key determinant of regional labor productivity [45]. Health level can also promote the prosperity of the local economy in various ways [46], and it is considered a component of human well-being [47]. Therefore, it deserves attention to guarantee the security and stability of the regional economy. Since regions with higher productivity can obtain more consumer markets, productivity is considered the key to determining the level of RES [29]. The secondary industry reflects regional productivity and is the main driver that promotes regional economic growth, especially in developing countries or regions. The tertiary sector can reflect the distribution of the regional economy in the social division of labor and economic functional departments and is the main force in ensuring regional economic stability and economic security.

The economic growth rate reflects the growth capacity of a region, especially the accumulation of human capital and fixed capital, two key components that enhance the potential and competitiveness of regional economies [40]. Stable economic growth can provide a better employment environment, create more products, and more social welfare. Investment in fixed assets is one of the decisive factors in a country/region’s ability to promote economic development. It constitutes the basic environment of regional economic operation, i.e., infrastructure, factories, roads, etc., in which lay the potential for regional economic growth [48]. In response to natural disasters, economic environment deterioration, and major external shocks, the regional economic foundation affects whether it can provide more diversified choices, thus improving local economic resilience and ensuring RES [49].

Thirdly, according to the institutional approach of economic geography, the market, institutions, and technology and their interaction constitute the dynamics of regional economic development so as to affect RES. The effective demand from enterprises, governments, consumers, import and export traders, and other actors affects the speed and quality of economic growth and also represents different sources of internal and external threats or crises affecting RES [50,51]. Markets can be divided into local (i.e., inside the region), external (i.e., the domestic market outside the locale), and foreign markets. The latter two markets can make up for a lack of local development capacities, such as cross-region or cross-border investment [24,30]. Foreign debt [23] can make up for a shortage of local economic development funds. Import trade can expand the market of local products, introduce new technologies and new demands, stimulate the upgrading of local production technologies, and improve the competitiveness of the local economy [30,33]. These market elements can enhance the strength and competitiveness of the local economy, but if there is excessive reliance on foreign markets or domestic external markets, a global economic crisis or a large-scale public health incident will reduce investment behavior, and the shrinking of the market will have a devastating impact on local RES. Therefore, identifying the potential of domestic and foreign markets of a region is crucial to evaluate RES.

The market mechanism does not always operate efficiently, and sometimes it brings about economic destruction due to its failure. Institutions, including governmental and non-governmental agencies, laws, regulations, values, routines, and other formal and informal institutions, can make up for such defects and facilitate the stable development of the regional economy. Institutional constraints affect the behavior of economic organizations, safeguard the interests of economic actors, avoid corruption, and prevent monopolistic behavior to improve the operational efficiency of the market. Technology, or innovation, not only serves as an economic factor and improves labor productivity but also creates new effective demands (e.g., new services and products) and increases sustainable supply [52]. In addition, another factor that frequently coincides with the instability of policies or institutions [53], political instability, is considered to have a profoundly negative impact on economic growth in general [54]. Due to its multidimensionality, there is no consensus on its definition and the appropriate number of these dimensions. Jong-a-Pin further examined such impacts by separating political instability into four dimensions, politically motivated violence, mass civil protest, instability within the political regime, and instability of the political regime, and found they have different effects on economic growth [55]. Political instability also affects the transformation of political institutions [53] and policy formulation and implementation [56]. As a consequence, in this paper, political stability is considered a driving force of RES in parallel with institutions in this border and ethnic region.

Technology innovation is beneficial in maintaining regional economic production and sustainability, as well as a key resource for a country/region to address future threats and challenges [57]. Moreover, it can break through the limitations of natural resource endowment and the ecological environment, realize the localized supply and trading of products, and reduce dependence on the external market.

According to the analysis above, the institutional approach of economic geography indicates the interaction of the three dimensions, RE, DF, and DF. It also provides the three dynamic mechanisms of economic spaces or places, namely, embeddedness, evolution, and differentiation, which are considered the result of the interaction among the three factors, the market, institutions, and technology, and decide the development path of economic spaces or RES [50]. The importance of the interaction between different factors and indicators concerning RES has already been realized; however, the extant literature has paid little attention to it [7].

## 3. Research Area, Indicator Selection, and Research Methods

### 3.1. Research Area

The Qinghai–Tibet Plateau mainly includes seven prefecture-level cities in the Tibet Autonomous Region, eight prefecture-level cities in Qinghai Province, eight prefecture-level cities in Sichuan Province, eight prefecture-level cities in Gansu Province, and four prefecture-level cities in the Xinjiang Uygur Autonomous Region. The latitude and longitude range of the Qinghai–Tibet Plateau is 26°00′12″ N–39°46′50″ N and 73°18′52″ E–104°46′59″ E. It is about 2800 km long from east to west, and 300 to 1500 km wide from north to south (Figure 1), with a total area of about 2.5 million square kilometers, making it the smallest population density in China. It is between 3000–5000 m above sea level, with an average altitude of more than 4000 m (Figure 2). The climate is characterized by strong radiation, abundant sunshine, and a large daily temperature difference. The average annual temperature decreases from 20 °C in the southeast to below −6 °C in the northwest. The water resources are mainly rivers, lakes, and glaciers. The main crops include highland barley and naked barley. The area below 3000 m in the southeast has better hydrothermal conditions, and rice can be planted. There are many ethnic minorities living in the region, including Tibetan, Hui, Naxi, and other ethnic groups. According to the statistics of prefecture-level cities, the GDP reached RMB 2048.123 billion in 2019, of which the tertiary industry accounted for 50.73%, and the population was 73,174,867. The per capita disposable income of urban residents and rural residents was RMB 30,435.21 and RMB 11,630.87, respectively.

### 3.2. RES Indicator Selection

#### 3.2.1. Basic Process

**Figure 4:**
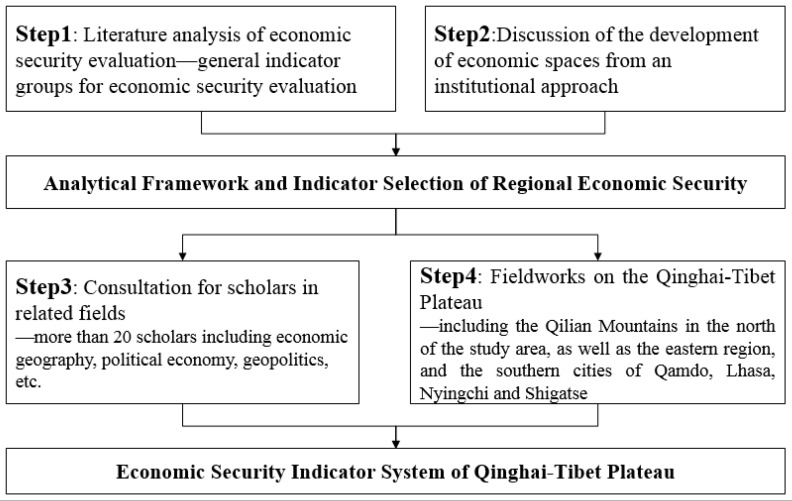
The Construction Process of RES Indicator System of Qinghai–Tibet Plateau.

According to the above triple-dimension analytical framework, the construction process of the RES indicator system (as shown in Figure 4) is implemented, which mainly includes the following steps.

The first step is to collate the common indicators and characteristic indicators of economic security assessment by comparing and analyzing the existing economic security research literature on different regional scales.

The second step is to use the institutional approach of economic geography on the formation and evolution of economic spaces or places and consider the suitability of extant assessment indicators of economic security/RES and regional differences and the neglect of dynamic mechanisms affecting RES, which has been neglected in extant literature; based on these, the indicator system of RES assessment is initially constructed.

The third step is to conduct a scientific investigation and research and revise and improve the initially constructed indicator system for the first time. From 2019 to 2021, the authors conducted fieldwork three times in the Qinghai–Tibet Plateau and investigated the differences in key factors concerning RES from different regional scales such as prefecture-level cities, counties, towns, villages, and farms. The main research areas were along the Qilian Mountains in the north, and Qamdo City, Lhasa City, Nyingchi City, and Shigatse City in the east and south.

The fourth step is to hold expert seminars in related fields to scientifically demonstrate and improve the first revised indicator system. In 2021, more than 20 experts and scholars with working experience in the Qinghai–Tibet Plateau in economic geography, political economy, geopolitics, and other disciplines attended 2 on-the-spot seminars and questionnaire surveys, etc., and the key indicators for evaluating the economic security of the Qinghai–Tibet Plateau were finally determined.

#### 3.2.2. Composition of RES Indicators

According to the construction process in Figure 4, 8 first-level indicators and 15 second-level indicators were selected from 3 aspects, i.e., resources and environment (RE), economic foundation (EF), driving forces (DF), and considering the availability of data (Table 2).

First of all, ecological protection is the bottom line for the economic security in the Qinghai–Tibet Plateau, and a good ecological environment is also the basic condition for achieving sustainable economic development in the Qinghai–Tibet Plateau. Therefore, in the economic development of the Qinghai–Tibet Plateau, ecological environment protection is the primary condition, and ecological conditions are an important part of the economic security assessment, which includes the following indicators.

(1) Vulnerability of ecological environment. The geographical characteristics of high altitudes in the Qinghai–Tibet Plateau and the influence of human activities determine the fragility of its ecological environment. This paper takes the fragility of the ecological environment of the Qinghai–Tibet Plateau as the basic condition that affects the economic security of the Qinghai–Tibet Plateau.

(2) Resources and economic factors. The labor force and grain production [58] are the basic resources and economic factors of regional economic development. Food security is an important part of national and regional economic security [59], and it is also one of the sustainable development goals of the United Nations [60]. Poor natural conditions, low grain output, and poor traffic in the Qinghai–Tibet Plateau lead to high transportation costs for external grain. According to our fieldwork, the dietary structure of the residents of the Qinghai–Tibet Plateau has changed; the demand for imported rice and wheat and local highland barley is on the rise, which increases the dependence of the food supply on other regions. Taking this into consideration, this article uses the regional food shortage index to measure local food security.

Per capita food demand is the key parameter for calculating regional food consumption and the food shortage index. The international standard for food security is not less than 500 kg of grain per capita per year (only the grain part is counted) [61,62,63]. The National Food and Nutrition Advisory Committee (2003) put forward the three-stage goal of per capita food security in China, specifically, a basic well-off society (2010), a well-off society in an all-round way (2020), and a transition period to prosperity (2030): 391 kg, 437 kg, and 472 kg, respectively. The Qinghai–Tibet Plateau is different from typical grain planting areas, and the food structure of residents is mainly dairy products and meat products. Therefore, the per capita food demand standard of animal husbandry or semi-farming and semi-animal husbandry areas should be lower than that of typical planting areas. According to the fieldwork, the per capita grain consumption demand of agricultural areas, semi-agricultural and semi-pastoral areas, and pastoral areas in the Qinghai–Tibet Plateau is 400 kg, 300 kg, and 200 kg, respectively. Considering the regional differences in the farming and animal husbandry system and residents’ dietary structure in the Qinghai–Tibet Plateau, a differentiated per capita food demand standard is formulated. The formula for calculating the food shortage index is:

(1) $$Q_{i}=\frac{O_{i}-C_{i}}{C_{i}}$$

where *Q_i_* represents the food shortage index of the *i*th region; *O_i_* represents the grain output of the *i*th county; *C_i_* represents the grain consumption of the *i*th county as measured by the per capita grain demand standard.

Secondly, indicators of the economic foundation include:

(1) Economic growth. The Qinghai–Tibet Plateau has a weak economic foundation and depends on financial support from the central government and counterpart support of the eastern provinces. Especially with the national strategy of “Western Development of China” since the late 1990s, the construction of infrastructure and the provision of public services has increased with a large number of investments, which stimulates economic growth and promotes local income levels. Therefore, this paper selects GDP growth rate and fixed asset investment growth rate as secondary indicators [23,50].

(2) Industrial structure. As mentioned in the introduction, secondary industry in the Qinghai–Tibet Plateau is underdeveloped and mainly relies on the development of local energy and mineral resources and a couple of industrial enterprises above the designated size. The Qinghai–Tibet Plateau has many unique natural and human landscape resources, and improved transportation conditions have stimulated the development of tertiary industries such as tourism, transportation services, catering, and hotels. This paper selects the proportion of industrial output value above the designated size and tertiary industry output value to GDP as indicators to represent the characteristics of the industrial structure of prefecture-level cities.

(3) Local affluence. Residents’ income level is an important manifestation of the local economic development level, and it may also increase local consumption and stimulate new economic growth [51]. Rural residents in the Qinghai–Tibet Plateau are mostly ethnic minorities. Their household income reflects the economic situation of ethnic minorities and is one of the factors affecting regional social stability and economic development. This paper selects the per capita income of urban residents and rural residents as two secondary indicators. Due to the data availability, instead of health expenditure, the number of local medical and health beds is used to measure the health level [47].

Thirdly, the indicators of development dynamics include:

(1) Market. The per capita retail sales of consumer goods are used to characterize the potential of the regional internal market. Foreign trade, as an external demand, is generally measured as investment [33], the proportion of FDI in GDP, export structure [29], foreign trade dependence [64], cross-border investment [24], etc. Due to data scarcity, the distance from border ports, instead of the indicators above, is used to measure the potential of foreign trade. The Qinghai–Tibet Plateau neighbors Pakistan, India, Nepal, and other countries/regions and also links China with western Asia and European countries through border ports, which increases the market opportunities for the region; this is a factor affecting the RES as well.

(2) Institutional and political stability. Fiscal transfer payments can address the problem of regional financial imbalance [65] and promote the stable development of the economy in backward areas, avoiding economic turbulence and promoting social stability [66]. The financial support from the central government is mainly used for infrastructure construction in the Qinghai–Tibet Plateau, such as the construction of the Qinghai–Tibet railway and expressway, public service facilities, such as hospitals and schools, and industrial infrastructure, such as industrial parks. Local fiscal expenditure can reflect the local government’s ability to provide basic public services and also indicates financial support from the central government. The higher the local fiscal revenue–expenditure ratio of the Qinghai–Tibet Plateau, the more it can reflect the financial dependence of the Qinghai–Tibet Plateau on the central government. Industrial parks, as geographical clusters of innovative factors, firms, other agencies, and institutions, can also reflect the support of other provinces or external enterprises as a response to the central government’s policy to firmly support the development of the Qing-Tibetan Plateau region. Therefore, the fiscal revenue–expenditure ratio and the number of industrial parks in the Qinghai–Tibet Plateau are used to measure the effects of formal policies and informal institutions on RES. As an ethnic border area, most local residents in the Qing–Tibetan Plateau region have religious beliefs. Meanwhile, religions are a main cause for hostile forces at home and abroad to instigate riots [67], and religious tensions harm political stability [55]. The central and local government has paid much attention to the policy of freedom of religious belief in this region and has strengthened the governance of public places for religious activities to keep a peaceful religious environment, which is crucial to local social and political stability. Therefore, this paper uses the number of places for religious activities per 10,000 people as an indicator of political stability.

(3) Technology. The labor force is considered an important factor in carrying technology and skills, and the quality of the labor force is an important indicator to reflect technical ability [64]. Most industrial enterprises above the designated size of the Qinghai–Tibet Plateau have been supported by the central or local government or other developed provinces. These companies and imported advanced production equipment and technology have significantly improved the labor productivity of the region. Therefore, this paper selects the number of industrial enterprises above the designated size and the number of students in ordinary schools and above as indicators to measure the technical level of regional productivity.

### 3.3. Data Sources

The data in this paper include socio-economic data, vector data, grid data, etc. Among them, the socio-economic data come from the statistical yearbooks of prefecture-level cities in the Qinghai–Tibet Plateau from 2000 to 2019, and the data on ecological vulnerability 1 km grid come from China National Qinghai–Tibet Plateau Scientific Data Center (http://data.tpdc.ac.cn/zh-hans/ accessed on 25 August 2022). Data on places for religious activities were obtained from the National Bureau of Religious Affairs website (www.sara.gov.cn/zjhdcsjbxx/index.jhtml/ accessed on 25 August 2022). The administrative boundary data and national boundary data of prefecture-level cities come from the standard map service network (http://bzdt.ch.mnr.gov.cn/ accessed on 25 August 2022), and the distances between prefecture-level cities and their nearest border ports were measured by ArcGIS software.

### 3.4. Research Methods

(1) Data standardization

In order to make the RES index in the range of 0–100, dimensionless, the selected indicators, the range standardization is used for standardization [32], and the formula is as follows:

(2) $$R_{ij}=\frac{x_{ij}-\min(x_{ij})}{\max(x_{ij})-\min(x_{ij})}$$

In the formula, the *x_ij_* represents *j*th indicator of the *i*th region, and the min (*x_ij_*) and max (*x_ij_*) represents the minimum and maximum values of the *j*th indicator.

(2) Entropy weight method

The entropy weight method is an objective weighting assessment method. The weight of assessment indicators depends on the variation degree of indicator value and the degree of variation between the max and min values, which eliminates the influence of subjective factors to some extent [68]. The relative importance of indicators is reflected by their variation degree. This paper uses the entropy weight method to measure the economic security level of prefecture-level cities in the Qinghai–Tibet Plateau in 2000 and 2019.

(3) RES index assessment model

The assessment model of a comprehensive RES index is calculated by using the weighted solution method, and the formula is as follows:

(3) $$W=\sum_{i}^{n}E_{i}\times X_{ij}$$

where *X_ij_* represents the specific index value, and *E_i_* represents the corresponding weight to each indicator. In order to keep the value of the comprehensive assessment index within 0–100, the indicators were standardized.

(4) Spatial autocorrelation analysis method

Spatial autocorrelation is one of the tools of spatial statistical analysis in geography, and its theoretical basis is the first law of geography; that is, similar things are more closely related [64]. Spatial autocorrelation is divided into global autocorrelation and local autocorrelation. Global autocorrelation is mainly used to analyze whether the overall distribution mode of spatial elements is cluster, random, or discrete distribution. The calculation results of global autocorrelation include Moran’s Index, Z score, and *p*-value. The higher Moran’s Index and Z score, the lower *p*-value, the more closely linked and the more clustered the elements are. This paper uses global autocorrelation to illustrate the overall spatial agglomeration of economic security in the research area. Local autocorrelation is the specific distribution pattern of analysis elements in space, that is, the high-value clustering area and the low-value clustering area of identification elements [65]. This paper uses it to explain the clustering degree of economic security in different areas and the high and low-value types of clustering.

## 4. RES Assessment in Qinghai–Tibet Plateau

This paper uses the entropy method to calculate the weight of each indicator of the three dimensions of RES, RE, EF, and DF, using the data of prefecture-level cities in the Qinghai–Tibet Plateau region in 2000, 2010, and 2019. On the whole, the weight value of the DF is greater than that of RE and EF (Table 3). Specifically, the weights of the number of industrial parks, the number of industrial enterprises above designated size, the number of students in ordinary schools and above, and the number of beds in medical institutions are much higher than those of other indicators, which predicts that institution, technology, and local affluence (health level) are crucial to guarantee local economic security.

### 4.1. Temporal and Spatial Differentiation of the Whole Region’s Economic Security

**Figure 5:**
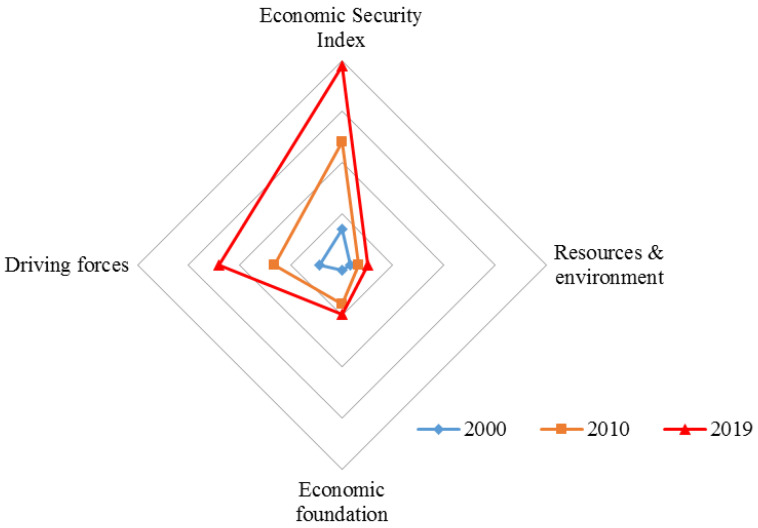
Radar Chart of Economic Security Index and Fractal Dimension Indexes of Qinghai–Tibet Plateau.

Using Formula (3), the comprehensive RES index of the research area in 2000, 2010, and 2019 is calculated, and the fracture dimension indexes of RE, EF, and DF are calculated, as shown in Figure 5.

The economic security of the Qinghai–Tibet Plateau is on the rise. The RES index increased dramatically from 14.04 in 2000 to 48.16 in 2010 to 77.69 in 2019. According to the fractal dimension indexes, RE and DF increased significantly, from 3.40 and 2.14 in 2000, 6.53 and 15.33 in 2010, to 10.22 and 19.40 in 2019, respectively, and have become the main dimensions promoting RES. As far as sub-indicators of RE, the proportion of the employed population increased from 15% in 2000 to 24% in 2019, which is helpful to increase residents’ income, stimulate consumption, and render the local market thriving. As far as sub-indicators of DF, as an indicator of institutions, the number of industrial parks increased by more than 2000 from 2000 to 2019, reflecting the improvement of the regional institutional environment and strengthening driving forces of regional economic development. Per capita retail sales (increasing from RMB 1481.93 in 2000 to RMB 15,705.67 in 2019), an indicator of regional market demand, and the number of students above middle school level (increasing from 316,329 in 2000 to 2,459,844 in 2019) and the number of industrial enterprises above designated size (increasing from 5165 in 2000 to 9787 in 2019), two indicators of regional technical level, also showed significant growth and jointly contributed to the improvement of RES.

However, the EF index shows a downward trend owing to the growth of investment in fixed assets, GDP growth, and the proportion of industrial output value above the designated size in GDP in 2019 decreased significantly compared with 2010.

The results of the global auto-correlation analysis are shown in Table 4. Generally, when the Moran’s I index is higher than 0, the Z score is higher than 1.65, and the *p*-value is less than 0.10, it belongs to the significant high-value clustering. According to the Z scores and the *p*-values in Table 4, Moran’s I index has an upward trend, increasing from 0.064892 in 2000 to 0.172454 in 2019, which indicates that the economic security of the Qinghai–Tibet Plateau shows a feature of increasing geographical agglomeration and spatial correlation. The economic security level of one prefecture-level city affects that of its neighboring cities; prefecture-level cities with a good foundation of economic security tend to conglomerate geographically and get better and better, while those with a poor economic foundation, resources, environment, and driving forces are always in a relatively poor situation. Consequently, the “Matthew effect (The Matthew effect refers to any individual, group or region, success and progress in a certain area can produce a cumulative advantage, and then can obtain more opportunities to achieve greater success. It mainly reflects the phenomenon of polarization and growing differences.)” may appear, and the difference in the economic security between cities with high levels and those with low levels would increase further.

### 4.2. Temporal and Spatial Differentiation of the Economic Security of Prefecture-Level Cities

**Figure 6:**
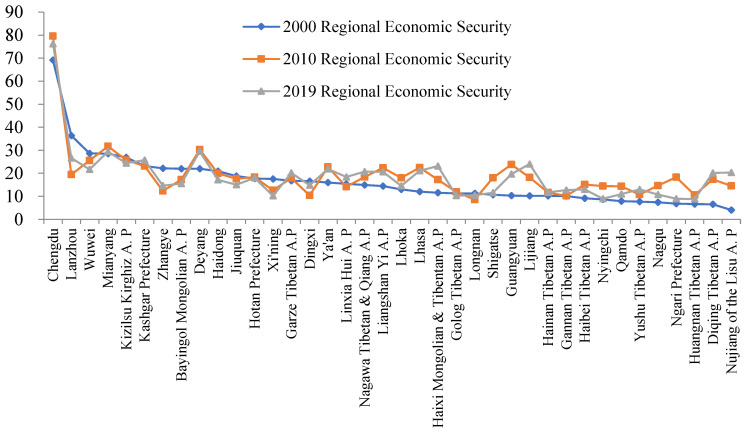

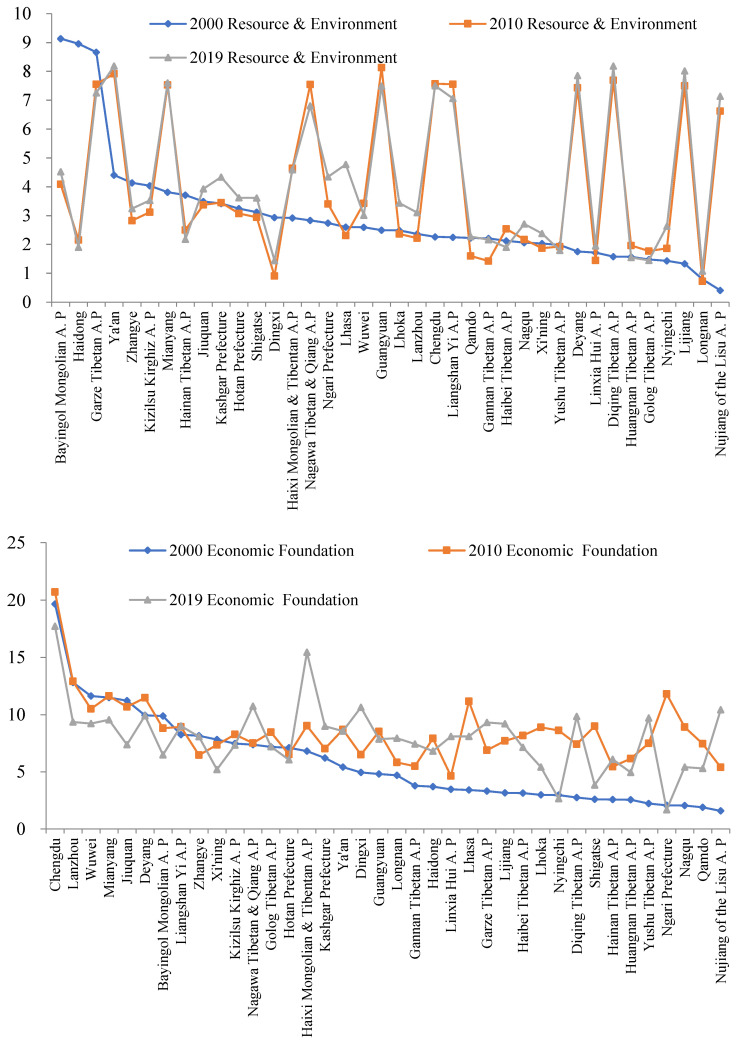

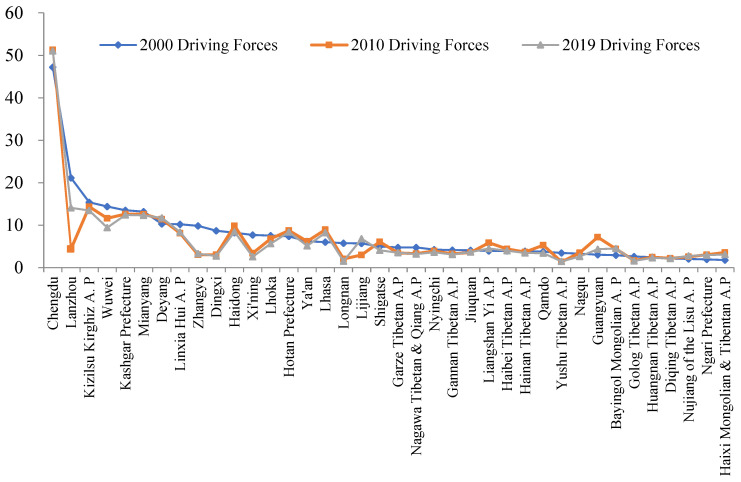
Economic Security Index and its Fractal Dimension Indexes of Prefecture-level Cities in the Qinghai–Tibet Plateau.

According to the economic security level of each prefecture-level city in 2000, from the highest to the lowest, the other two curves of the economic security of cities in 2010 and 2019 were made (Figure 6). The figure shows that the RES curve in 2010 is similar to 2019, and there is a significant change in 2000, which means that the economic security of prefecture-level cities changed greatly from 2000 to 2010, and then was stable afterward. It also presents path dependency, that is, the economic security level of a city in the early stage may affect its changes in the later stage, which consequently shows a tendency that “the strong are always strong, while the weak are always weak”. Moreover, there are fluctuations in some parts of the curves, mainly in the higher level parts and the lowest level parts. Some former cities improved their economic security during this period, such as Haixi Mongolian Autonomous Prefecture, Ya’an, Guangyuan, Diqing Tibetan Autonomous Prefecture, Lijiang, Lhasa in Tibet and Garze, Nagawa, and Liangshan prefectures; while the latter cities have significantly decreased in RES in 2019, compared with 2010, such as Lanzhou, Wuwei, Zhangye in Gansu province, and Nagqu, Shigatse, Qamdo, Ngari Prefecture, Nyingchi in Tibet Autonomous Region.

From the changes in the fractal dimension indexes (Figure 6), it can be found that compared with those in 2000, the EF index and RE index of most prefecture-level cities in 2019 has not been significantly changed. The majority of prefecture-level cities have a low level of DF, except for a couple of cities, such as Chengdu, Mianyang, and Deyang in Sichuan Province, which are close to the Chengdu–Chongqing urban agglomeration, with ample market demand, advanced technology, and open and innovative institutions, which leads to their relatively high economic security. The situation and change of Fractal Dimension Indexes of economic security led to the change and spatial differentiation of the economic security of prefecture-level cities in the Qinghai–Tibet Plateau. Among them, the RES decreased significantly in cities such as Lanzhou, Wuwei, Zhangye, Bayingol, and Xining. City EF decreased significantly, and RES increased significantly in cities such as Lhasa, Guangyuan, and Lijiang. City EF has an obvious upward trend; the improvement of RES in Chengdu and Qamdo is mainly due to the rise of RE, and it can be seen that the change of EF has a great impact on the change of RES.

**Figure 7:**
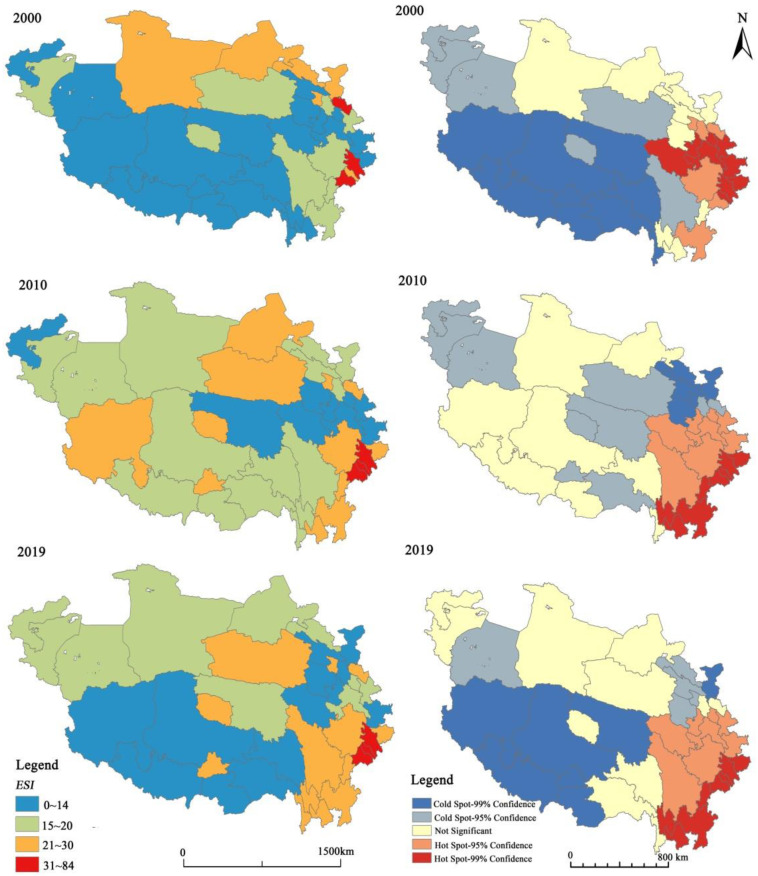
Spatial Pattern (**Left**) and Local Spatial Cluster (**Right**) Map of the Economic Security of Prefecture-level Cities in the Qinghai–Tibet Plateau.

According to the hot spot analysis chart of local spatial autocorrelation (Figure 7), the areas with high RES and low RES are geographical clusters. The economic security of prefecture-level cities in the Qinghai–Tibet Plateau presents a spatial pattern of “high edge and low center”, namely, prefecture-level cities on the edge of the plateau have a high level of RES, while those in the center area have a low level of RES in general. The high-value areas tend to shift from the northeast to the southeast, while the low-value areas are relatively stable, mainly concentrated in Tibet, Xinjiang, Gansu, and other regions. In particular, prefecture-level cities such as Shigatse, Nagqu, Lhoka, and Ngari Prefecture in Tibet Autonomous Region have always been significant low-value clusters, while Garze in Sichuan Province, Lijiang, and Diqing in Yunnan Province changed from low-value clusters and intermediate areas in 2000 to high-value clusters in 2019.

The coefficient of variation is used as an indicator to further present the spatial differences in the economic security of prefecture-level cities in the Qinghai–Tibet Plateau. According to the result (Table 5), the coefficient of variation of the RES index in the Qinghai–Tibet Plateau is still at a high level (a variable coefficient above 0.5 indicates a significant difference), but from the coefficient of variation of the economic security index and its fractal dimension indexes, the spatial coefficient of variation of the economic security index has a downward trend, which means the spatial differences are significant but tend to shrink. The coefficient of variation was the highest in 2000, and the gap between prefecture-level cities was the highest in that year. The coefficients of variation in 2010 and 2019 were lower than 0.60, indicating that there was a downward trend from 2000 to 2019. The variation coefficients of the fractal dimension indexes, RE and EF, also have a downward trend, especially for that of the economic foundation index. Its dramatic decrease shows that the regional heterogeneity of the economic development base became relatively low in 2010 and 2019. This is probably because the differences between cities in residents’ living standards, economic growth rate, and industrial structure have been narrowing. However, the coefficient of variation of the DF index is increasing greatly, which shows that driving forces of regional economic development exert an increasing effect on the regional heterogeneity of the cities’ RES. From the variation trend of the coefficient of variation, regional economic security is consistent with RE and EF, and they are opposite to DF, which implies that some cities with higher levels of RES in the previous years encountered a lack of driving forces in recent years, while other areas with lower levels of RES in the previous years have increased in driving forces lately.

### 4.3. Classification of RES Types

#### 4.3.1. Classification of RES Types

**Figure 8:**
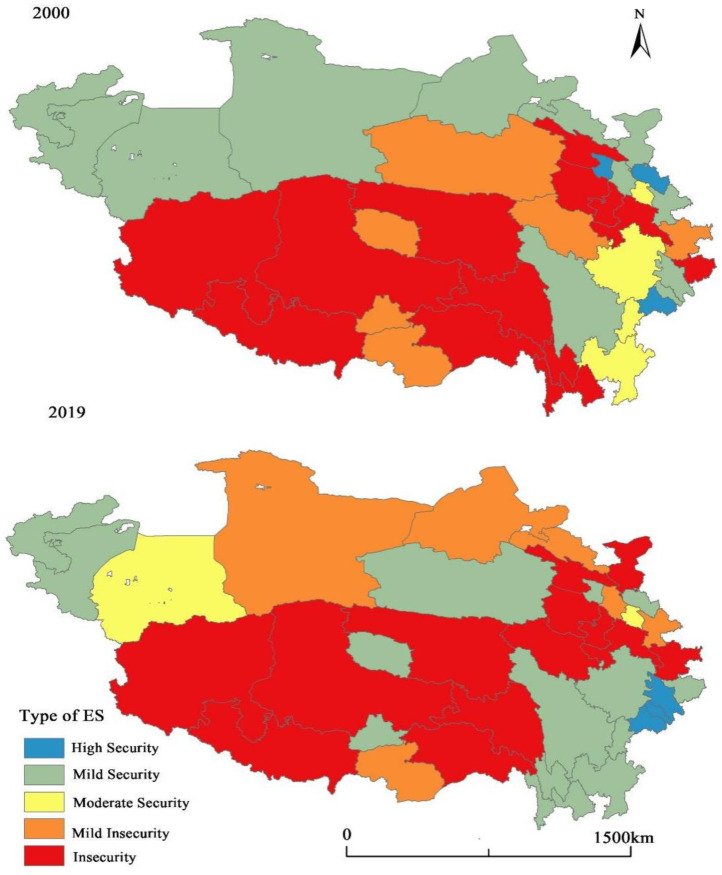
Spatial Pattern of Economic Security Types of Prefecture-level Cities in the Qinghai–Tibet Plateau in 2000 and 2019.

This paper further defines the median and average of the economic security index as the safety boundary points and defines the top 20% higher than the average as the high safety zone and the middle safety zone between the average and the median. The first 20% below the median is defined as mild insecurity, and the rest is unsafe. Therefore, the types of economic security in the Qinghai–Tibet Plateau are divided into five types: high security, medium security, mild security, mild insecurity, and insecurity (as shown in Figure 8).

As mentioned above, the economic security type of the Qinghai–Tibet Plateau is geographically characterized by “low center and high edge”, that is, the prefecture-level cities in the edge area are relatively safe in terms of RES, while the core area is mostly in a slightly unsafe state. Basically, the spatial pattern of the economic security of the Qinghai–Tibet Plateau is consistent with its general topography. It can be seen that the economic security types of the areas along the Kunlun Mountains, Himalayan Mountains, Gangdise Mountains, Tanggula Mountains, Bayankala Mountains, Qilian Mountains, etc., are mostly unsafe and slightly unsafe, while the economic security types of peripheral areas and basin areas are relatively safe. Generally, the areas along tall mountains have some disadvantages in terms of fragile ecological environment, high transportation costs, and economic costs, and these areas are sparsely populated and have a weak foundation for economic development. Moreover, they are highly vulnerable to economic risks in the face of major natural disasters and external shocks.

The economic security in the Qinghai–Tibet Plateau has generally turned to the state of safety, but the central areas still have difficulty improving economic security except for a few cities, such as Lhasa. The safe, stable, and safe areas are mostly located in the marginal areas of the Qinghai–Tibet Plateau, especially in the southeastern marginal areas.

Areas with relatively safe economies make up the majority. According to Figure 8, six cities located in the marginal areas of the Qinghai–Tibet Plateau have been in a stable and safe state for a long time, including Chengdu, Ganzi Tibetan Autonomous Prefecture, Linxia Hui Autonomous Prefecture, Kizilsu Kirghiz Autonomous Prefecture, and Kashgar Prefecture. These cities have good economic foundations, market conditions, and labor resources, and relatively good resources, ecological environment, and transportation conditions. The other five cities located on the northern edge of the Qinghai–Tibet Plateau, including Dengyang City, Mianyang City, Ya’an City, Nagawa Tibetan and Qiang Autonomous Prefecture, and Liangshan Yi Autonomous Prefecture in Sichuan Province, are also in a safe state despite their RES reducing. Six cities located on the southeast edge have changed from unsafe to safe, including Guangyuan City, Diqing Prefecture, Nujiang Prefecture, Haixi Prefecture, Lhasa City, Lijiang, etc. Sichuan Province and Yunnan Province have relatively good economic foundations and natural environments, and China’s “Precision Poverty Alleviation Plan” has successfully promoted the economic development of Liangshan Prefecture, Diqing Prefecture, and Nujiang Prefecture [69]. Haixi Prefecture is located in the Qaidam Basin, and China’s “Western Development Strategy” for decades has stimulated the development of mineral resources and new energy and accelerated the industrialization process in this area [70]. As the capital of the Tibet Autonomous Region, Lhasa has a high administrative level. The central government’s support has promoted the development of Lhasa in many aspects, such as industrial revitalization, labor training, technology introduction, and capital investment.

**Figure 9:**
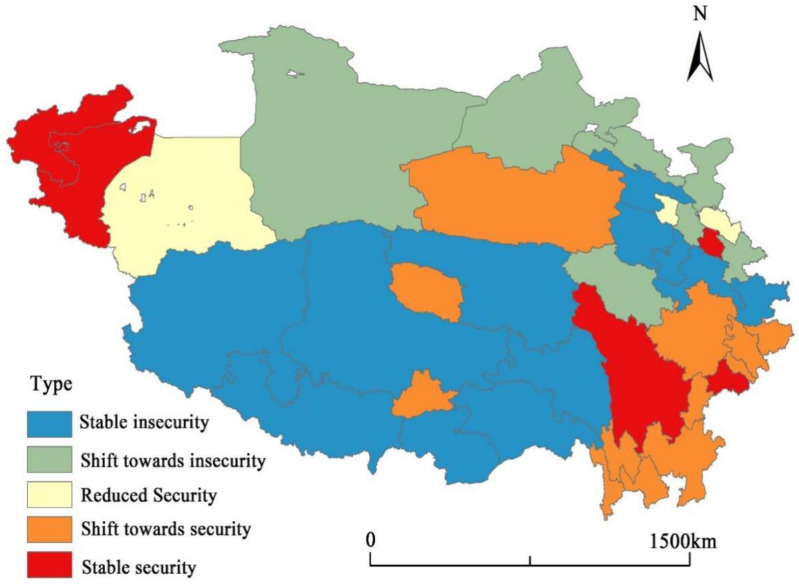
Transformation of the Economic Security Types of Prefecture-level Cities in Qinghai–Tibet Plateau in 2000 and 2019.

Economically insecure areas still face enormous challenges. Twelve cities across the northeast to the southwest area of the Qinghai–Tibet Plateau have maintained a state of long-term economic insecurity (Figure 9). Seven cities in the northern and eastern areas have changed from a state of mild security to mild insecurity and insecurity. Further analysis shows that most of these cities are dominated by traditional extractive industries and resource-based industries, and they have been facing pressure from the decline of traditional industries, the outflow of population, and decreased investment. Moreover, the fragile ecology and alpine environment in the plateau area lead to poverty, endemic diseases, and poor health, which have a negative impact on the local labor market, such as few job opportunities, low productivity, and decreasing income levels. Additionally, the border cities in Tibet have maintained a state of economic insecurity for a long time, probably because the border disputes between China and India have hampered the economic development of these cities. By contrast, the border cities in Xinjiang have been in a state of economic stability and security because the long-term strategic alliance between China and Pakistan has sustained the economic development of these cities.

#### 4.3.2. Analysis of Fractal Dimension Indexes of RES

This subsection further analyzes the RES from three dimensions: RE, EF, and DF, with the method of multidimensional feature coupling. Taking the average value of each dimension index as the demarcation point, the three dimensions are individually divided into two levels: high value and low value. Consequently, they are combined to be divided into eight types, as shown in Table 6, which represent eight mechanisms affecting RES. According to this table, Type 1, with the highest level of three dimensions of RES, has a few prefecture-level cities and slightly increases in the number of prefecture-level cities, while type 8, with the lowest level of three dimensions of RES, has the most number of prefecture-level cities, being 2–3 times than Type 1.

**Figure 10:**
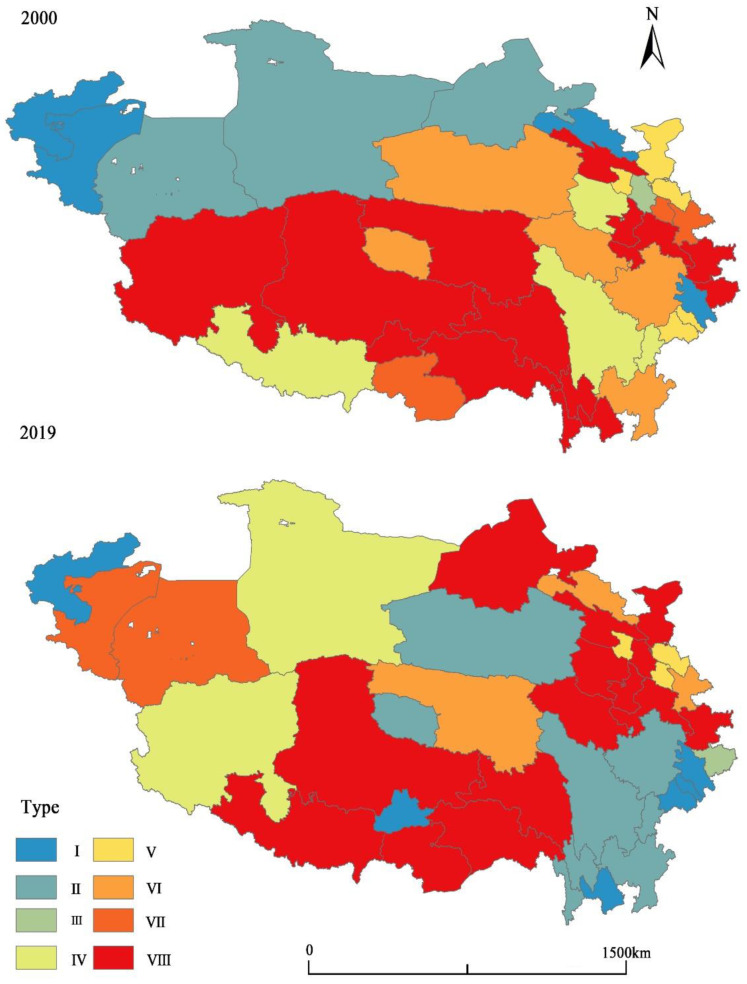
Spatial Pattern of Fractal Dimension Indexes Types of the Economic Security in the Qinghai–Tibet Plateau in 2000 and 2019.

Figure 10 shows that Type 2, Type 6, and Type 8, having something in common in the low level of driving forces, are the main types with an increasing trend, and these three types of prefecture-level cities are spatially clustered and contiguous in 2019 and 2000, though the specific prefecture-level cities in each type vary considerably. Among them, Type 8 occupied a large proportion in both 2000 and 2019, and this type of prefecture-level city was mainly distributed in the Tibet Autonomous Region. It shows that many prefecture-level cities in this region still face serious problems of fragile ecology, scarcity of economic factors, weak economic foundation, and driving forces. Type 6 prefecture-level cities are mainly distributed in Qinghai and Gansu Provinces, and Type 2 prefecture-level cities have ‘shifted’ from the northern edge to the southeastern edge of the Qinghai–Tibet Plateau.

In terms of the fractal dimensions of RES, the prefecture-level cities of Type 2, Type 6, and Type 8 have few industrial enterprises above the designated size and few industrial parks, which means technology and institutions, two important driving forces, cannot provide enough support for the economic development of cities. If compared with the regions of Type 1 with a high level of driving forces, such weakness of these two types seems striking. In 2019, the number of industrial enterprises above the designated size in Chengdu, Deyang, and Mianyang with the highest security index was more than 1000–3000, while the number of regions with the lowest security index was about 100. These three main types demonstrate that the weakness of economic driving forces is negatively affecting the economic security of the Qinghai–Tibet Plateau, followed by resources and economic factors and ecological environment conditions.

Type 3, Type 4, Type 5, and Type 7 are relatively few and scattered in the eastern edge area. The three latter types, Type 4, Type 5, and Type 7, decreased in the number of prefecture-level cities in 2000 and 2019.

## 5. Conclusions and Discussion

### 5.1. Conclusions

This paper uses the economic geography approach to construct the analytical framework of RES assessment from three dimensions, resources and environment (RE), economic foundation (ED), and driving forces (DF), and illustrates the interaction among these three dimensions and their joint effects on RES. This not only makes up for the deficiency in the extant literature, which pays little attention to regional characteristics in indicator selection and the dynamic mechanism concerning RES [36], but also enriches case studies of different regional scales, providing a feasible analytical framework for RES research. Moreover, it emphasizes institutional and political stability and market and technology as a driving force, which provides a feasible tool for policy-making to improve their RES level, especially for underdeveloped border regions such as the Qinghai–Tibet Plateau in China, a typical ethnic region. Additionally, it introduces health level as an indicator of RES, which suggests a new thought and concerns about the development of local residents, not only because they provide labor as an economic factor, but because the improvement of their educational level and health level increases the level of local human capital so as to improve productivity, resist risks, and strengthen regional economic security.

This paper makes the following specific conclusions. (1) The index of the economic security in the Qinghai–Tibet Plateau is on the rise, and the difference in the level of RES among cities is significant but tends to decrease. Specifically, Chengdu, Guangyuan, and Lijiang in Sichuan Province, the southeastern area, and Lhasa in Tibet tend to increase in RES, while Lanzhou, Wuwei, Zhangye, and other cities in the northern Gansu Province have a downward trend. (2) There is a significant spatial autocorrelation among cities in the Qinghai–Tibet Plateau in terms of RES, which shows that areas with the same or similar levels are geographically concentrated and contiguous, and a city’s RES is affected by its neighboring cities. The high-value areas are concentrated along the southeast edge, and the low-value areas are concentrated in the central and southwest areas. (3) In spite of lower weight values, the weakness of the economic foundation and the fragility of the ecological environment has increasingly hampered the improvement of the economic security in the Qinghai–Tibet Plateau. In terms of driving forces, it is the support of the central government and the aid programs of other provinces (by increasing financial transfer payments, building industrial parks, introducing enterprises, and other measures) that contributes to the economic development of the Qinghai–Tibet Plateau, which also suggests that these support policies and political stability are crucial to guarantee the economic security of the Qinghai–Tibet Plateau.

### 5.2. Discussion

There are some limitations to this paper. Firstly, the assessment framework of RES only attempts to analyze the impact of RE, EF, and DF on RES but does not deeply discuss their interaction and mechanism. In the future, more discussion about this point is needed, for example, examining the interaction of market, institutional and political stability, and technology in terms of embeddedness, differentiation, and evolution [51] and their effects on RES. Secondly, the incomplete statistical data of the Qinghai–Tibet Plateau decreased the representation of some indicators. One example is using the distance of a prefecture-level city from its nearest border port to represent the dependence on the foreign market due to incomplete trade data for imports and exports and data for foreign direct investment. In the future, it is expected that big data or other reliable survey databases will be used to improve this assessment system further. For example, online public opinions or questionnaire surveys can be collected and constructed as indicators of policy stability. Thirdly, globalization has had a profound impact on countries and their inner urban areas and has provided new perspectives and approaches to economic security [71]. Factors in the global dimension, such as global financial market instability, global energy price fluctuations, foreign trade flows, changes in foreign direct investment, and global climate trends, deserve attention. Last but not least, more quantitative comparative studies, such as fuzzy assessment and analytic hierarchy process, are needed to verify the reliability of the results of this research.

## Figures and Tables

**Table 1:** Assessment Indicators of Economic Security Literature.

Author(s)	Year	Country/Region	Purposes/Contributions	Main Ideas	ES Indicators	Method
Ignatov et al. [23]	2019	Cross-border areas in the EU	To assess the extent to which the economic security of the European Union has changed in the period from 2007 to 2017.	Key elements of economic security or the threats of major economies are based on the actual situation and depend on the specific environment by which each country is characterized.	GDP growth, debt, fixed capital investment, productivity, technology, institutional performance	Qualitative and quantitative analyses of data
Gryshova et al. [2]	2020	Cross-border regions in Ukraine and the EU	To compare the economic security gap between Ukraine and the EU and to verify the hypothesis that economic security affects regional sustainable development.	The economic security of a country is influenced by threats that manifest themselves in all spheres of public life, including economic, political, social, and environmental ones.	Global competitiveness index, globalization index, fragile states index, Legatum prosperity index, human development index, and environmental performance index	Geometric mean, cluster analysis, linear regression
Lee [24]	2021	Cross-border areas in Southeast Asia	To examine the evolving nature of ASEAN’s economic security with a focus on regional economic initiatives.	Increased inter-connectivity based on institutional frameworks has allowed ASEAN countries to enhance security in a traditional sense.	Cross-border investment, cooperation, depth of agreements, flexibility of agreements	Qualitative analyses
Kravchenko et al. [22]	2021	Sub-national regions in Russia	To develop a universal method for assessing threats to the economic security of the region.	Public procurement is one of the most important elements of the economic security system of a region.	The quantity of bidding, the cost of bidding, the average contract price, the proportion of local procurement from SMEs, and the number of suppliers participating in bidding	Least square method and cluster analysis
Onyshchenko and Bondarevska et al. [8]	2018	Sub-national regions in Ukraine	To develop a methodology for assessing the economic security of the region on the basis of the analyzed basic methods and techniques.	Based on the fundamental provisions of economic theory, modern concepts of economic security on the mesolevel, statistical indicators describe the current state of the economy of the region and threats to regional security.	Financial security, social security, investment, and innovation security, foreign economic security, and population security	Integral formula
Lefimova, Labartkava, Pashchenko et al. [27]	2020	Sub-national regions in Ukraine	To formulate a methodological framework for assessing the economic security of the region’s development.	The indices of economic security characterize the achieved level of economic development of the region and the preconditions of further activity.	Total added value, total import and export volume, consumer price index, investment activity level, total investment, investment growth rate, infrastructure development level, credit and debt structure, population size, labor force, average wage	Integral and weighted solution method
Arkhipova and Kulikova et al. [28]	2020	Sub-national regions in Russia	To assess the level of the innovative development of the Volga Federal District and identify zones of relative stability, medium, and critical state.	Innovation can improve the efficiency of major economic activities in a certain range and ensure economic security.	Index of economic innovation components, comprehensive innovation index, and innovation development index	No details
Olha Ovcharenko et al. [13]	2022	Sub-national regions in Ukraine	To form a methodological tool for RES assessment based on the fuzzy modeling method and to develop a method for public departments and local governments to manage RES.	Based on extant studies, the major components of economic security of a region are the investment, innovation, financial, foreign trade, demographic, social security, and security of economic activity.	The ratio of capital to GRP, innovative activities, financial security, the proportion of imports and exports, the proportion of imports to GRP, the unemployment rate, the proportion of people with incomes below the level of food and clothing, the overall crime rate, the income level of people, the agricultural production index, the industrial production index, and the GRP volume ratio	Fuzzy logic method

**Table 2:** RES Indicator System of Qinghai–Tibet Plateau.

Target Layer	Primary Indicators	Secondary Indicators
RE	Ecological environment	Ecological environmental vulnerability ^1^
	Resources and economic factors	Food security index
		The total number of employed people as a proportion of the total population
EF	Local affluence	Per capita income of urban residents
		Per capita income of rural residents
		Number of beds in hospitals and health centers
	Industrial structure	Proportion of industrial output value above designated size
		Proportion of output value of tertiary industry
	Economic Growth	GDP growth rate
		Investment growth in fixed assets
DF	Market	Reverse of the distance from the nearest border port
		Per capita retail sales of social consumer goods
	Institution & Political stability	Ratio of fiscal expenditure to fiscal revenue
		Number of industrial parks
		Number of places for religious activities per 10,000 people
	Technology	Number of students in ordinary schools and above
		Number of industrial enterprises above designated size

^1^ Based on the vulnerability assessment framework of “exposivity (selecting the population density, the density of livestock, the density of road network, density of settlements as sub-indicators), sensitivity (average annual temperature, average annual precipitation, at an altitude of slope, soil sand content, soil organic matter ), and adaptability (net primary productivity, vegetation coverage, index of biodiversity)”, the vulnerability assessment index system of agricultural and pastoral areas on the Qinghai–Tibet Plateau was constructed. Yaahp software (version 10.3) was used to establish the analytic hierarchy process model, and the judgment matrix was established based on the questionnaire survey of six experts in related fields. The data passed the consistency test (consistency: 0.0000), and the weight of each indicator was obtained (TENG Yanmin, ZHAN Jinyan, LIU Shiliang. A 1 km grid dataset of ecological vulnerability in agricultural and pastoral areas of Qinghai Tibet Plateau. National Tibetan Plateau Data Center, DOI:10.11888/Ecolo.tpdc.271117, CSTR:18406.11.Ecolo.tpdc.271117, 2021).

**Table 3:** Weight of Each Secondary Indicator Calculated by Entropy Weight Method.

Target Layer	Primary Indicators	Secondary Indicators	Weight
RE (resources and environment)	Ecological environment	Ecological environmental vulnerability	0.026
	Resources and economic factors	Food security index	0.008
		The total number of employed people accounts for the total population	0.075
EF (economic foundation)	Local affluence	Per capita income of urban residents	0.038
		Per capita income of rural residents	0.047
		Number of beds in hospitals and health centers	0.094
	Industrial structure	Proportion of industrial output value above designated size	0.039
	Growth rate	Proportion of output value of tertiary industry	0.015
		GDP growth rate	0.035
		Growth rate of investment in fixed assets	0.015
DF (driving forces)	Market	Reverse of the distance from the nearest border port	0.052
		Per capita retail sales of social consumer goods	0.047
	Institutional and political stability	Ratio of fiscal expenditure to fiscal revenue	0.008
		Number of industrial parks	0.172
		Number of places for religious activities per 10,000 people	0.085
	Technology	Number of students in ordinary schools and above	0.122
		Number of industrial enterprises above designated size	0.123

**Table 4:** Global Spatial Autocorrelation of Economic Security in the Qinghai–Tibet Plateau.

	Moran’s Index	Z-Score	*p*-Value
2000	0.064892	1.710063	0.087254
2010	0.176277	4.313678	0.000016
2019	0.172454	4.056822	0.000050

Note: the Z score is the standard deviation multiple of the variable in each prefecture city, while the *p*-value indicates the probability that the distribution pattern is randomly generated.

**Table 5:** Average, Standard Deviation and Coefficient of Variation of Economic Security and its Fractal Dimension Indexes of Prefecture-level Cities.

Index Name	Year	Average Value	Standard Deviation	Variable Coefficient
RES	2000	16.392	11.432	0.697
	2010	19.196	11.45	0.596
	2019	18.799	11.238	0.598
RE	2000	2.983	1.979	0.663
	2010	3.927	2.478	0.631
	2019	4.226	2.355	0.557
DF	2000	5.868	3.922	0.668
	2010	8.53	2.82	0.331
	2019	7.997	2.982	0.373
DF	2000	7.541	7.932	1.052
	2010	6.738	8.189	1.215
	2019	6.576	8.225	1.251

**Table 6:** Types of Fractal Dimension Indexes of the Economic Security in the Qinghai–Tibet Plateau.

	RE	EF	DF	2000ESI	2019 ESI
Type Ⅰ	H	H	H	Mianyang, Zhangye, Kashgar Prefecture, Kizilsu Kirghiz Autonomous Prefecture	Chengdu, Deyang, Lhasa, Kizilsu Kirghiz Autonomous Prefecture, Mianyang, Lijiang
Type Ⅱ	H	H	L	Bayingol Mongolian Autonomous Prefecture, Hotan Prefecture, Jiuquan	Nagawa Tibetan and Qiang Autonomous Prefecture, Diqing Tibetan Autonomous Prefecture, Garze Tibetan Autonomous Prefecture, Haixi Mongolian and Tibentan Autonomous Prefecture, Liangshan Yi Autonomous Prefecture, Nujiang of the Lisu Autonomous Prefecture, Ya’an
Type Ⅲ	H	L	H	Haidong	Guangyuan,
Type Ⅳ	H	L	L	Garze Tibetan Autonomous Prefecture, Hainan Tibetan Autonomous Prefecture, Shigatse, Ya’an	Bayingol Mongolian Autonomous Prefecture, Ngari Prefecture
Type Ⅴ	L	H	H	Chengdu, Deyang, Lanzhou, Wuwei, Xi’ning	Lanzhou, Xi’ning, Linxia Hui Autonomous Prefecture
Type Ⅵ	L	H	L	Nagawa Tibetan and Qiang Autonomous Prefecture, Golog TibetanAutonomous Prefecture, Haixi Mongolian and Tibentan Autonomous Prefecture, Liangshan Yi Autonomous Prefecture,	Dingxi, Yushu Tibetan Autonomous Prefecture, Zhangye
Type Ⅶ	L	L	H	Dingxi, Lhoka, Linxia Hui Autonomous Prefecture,	Hotan Prefecture, Kashgar Prefecture
Type Ⅷ	L	L	L	Diqing Tibetan Autonomous Prefecture, Gannan Tibetan Autonomous Prefecture, Guangyuan, Haibei Tibetan Autonomous Prefecture, Huangnan Tibetan Autonomous Prefecture, Lhasa, Lijiang, Nagqu, Ngari Prefecture, Nujiang of the Lisu Autonomous Prefecture, Nyingchi, Qamdo, Yushu Tibetan Autonomous Prefecture, Longnan	Gannan Tibetan A.P, Golog Tibetan Autonomous Prefecture, Haibei Tibetan Autonomous Prefecture, Hainan Tibetan Autonomous Prefecture, Huangnan Tibetan Autonomous Prefecture, Longnan, Haidong, Jiuquan, Lhoka, Nagqu, Nyingchi, Qamdo, Shigatse, Wuwei

## Data Availability

The data used in this paper include socio-economic indicators, vector data, grid data, etc. Among them, the data on socio-economic indicators used to establish the indicator system comes from the statistical yearbooks of prefecture-level cities in Qinghai–Tibet Plateau from 2000 to 2019, and the data on ecological vulnerability 1km grid comes from the China National Qinghai–Tibet Plateau Scientific Data Center (http://data.tpdc.ac.cn/zh-hans/ accessed on 25 August 2022). The administrative boundary data and national boundary data of prefecture-level cities come from the standard map service network (http://bzdt.ch.mnr.gov.cn/ accessed on 25 August 2022).
